# Correction to: TRIM29 facilitates the epithelial-tomesenchymal transition and the progression of colorectal cancer via the activation of the Wnt/β-catenin signaling pathway

**DOI:** 10.1186/s13046-021-01922-w

**Published:** 2021-04-28

**Authors:** Juntao Sun, Tianyu Zhang, Mengmeng Cheng, Liwen Hong, Chen Zhang, Mengfan Xie, Peijun Sun, Rong Fan, Zhengting Wang, Lei Wang, Jie Zhong

**Affiliations:** Department of Gastroenterology, Ruijin Hospital, Shanghai Jiao Tong University School of Medicine, Shanghai, 200025 China

**Correction to: J Exp Clin Cancer Res 38, 104 (2019)**

**https://doi.org/10.1186/s13046-019-1098-y**

Following the publication of the original article [1], the authors identified minor errors in image-typesetting in Fig. 2, Fig. 6 and Fig. 7; specifically panels Fig. 2h, Fig. 6d and Fig. 7c. The specific panels that have been corrected are as follows:

- Fig. 2h: all migration and invasion panels
- Fig. 6d: all panels
- Fig. 7c: both XAV939(+) panels

**Fig. 2:**
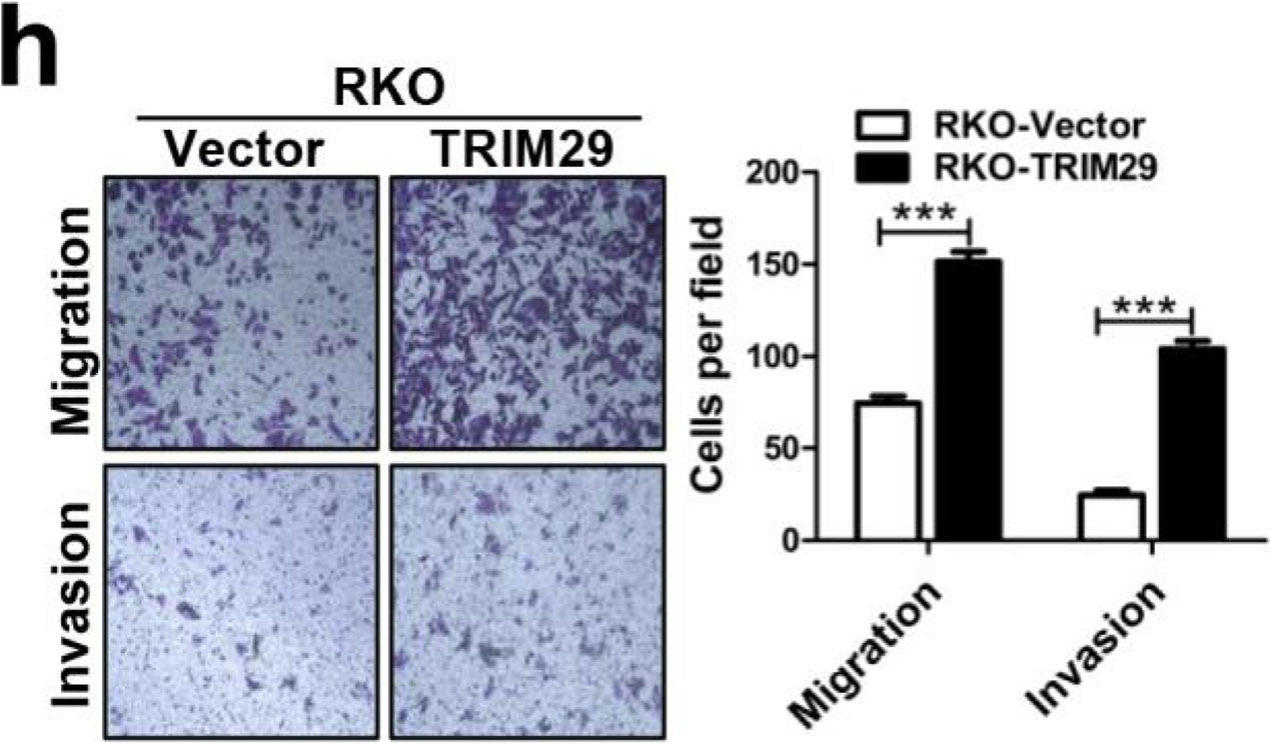
**h** The migration and invasion assays showed different cell motilities in modified RKO cells. Overexpression of TRIM29 promoted the migration and invasion of RKO cells. All of the data are presented as the mean ± SEM. from three independent experiments (***P < 0.001)

**Fig. 6:**
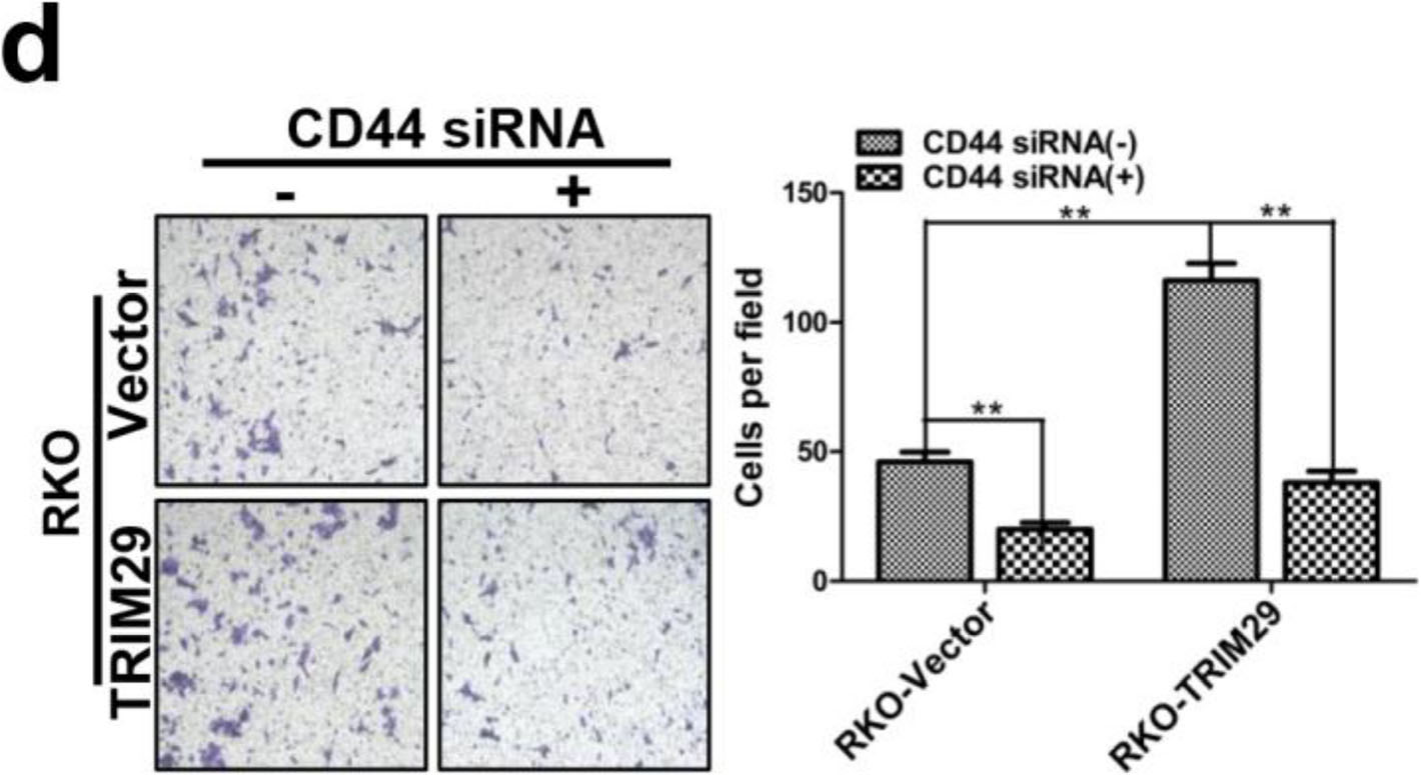
**d** The Transwell invasion assay showed different cell invasive abilities in RKO-Vector and RKO-TRIM29 cells which were transfected with negative control siRNA or siRNA against all CD44 isoforms. The data are presented as the mean ± SEM from three independent experiments (**P < 0.01)

**Fig. 7:**
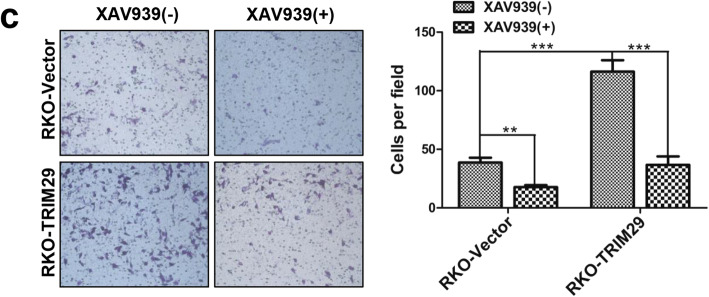
**c** The Transwell invasion assay showed different cell invasive abilities in RKO-Vector and RKO-TRIM29 cells with XAV939 (15 μM) for 24 h. The data are presented as the mean ± SEM. from three independent experiments (**P < 0.01, ***P < 0.001)

The corrected figures are provided below. The corrections do not have any effect on the results or conclusions of the paper. The original article has been corrected.
